# Opinions of the Press

**Published:** 1887-11-19

**Authors:** 


### THE NATIONAL PENSION FUND.

### OPINIONS OF THE PRESS.

### Pall Mall Gazette.

If there is any truth in the recent announcement that the Queen will devote the Women's Jubilee Offering for the benefit of nurses and nursing institutions, it is sincerely to be hoped that her Majesty will look with favour upon the excellent scheme for providing a National Pension Fund for certificated nurses and accredited hospital officials. The fund must be so organised as to be entirely self-supporting—that is, the plan must be such as to ensure permanency and solvency, apart altogether from any donations which may be received from non-members. Everybody knows that the work of nurses of the sick is but poorly paid at the best, and that it is indeed work which never can be adequately paid for, and it is just for this reason that the present scheme deserves the more sympathy, as everyone of the members contributes his or her own mite to the fund, and that indeed it will be self-supporting, the only difference being that in case the Queen contributes, or, failing this, donations are made towards it by the public, the provision for the members will be more ample, and the project can be worked without delay.

### Birmingham Daily Post.

Of women who devote themselves to nursing, the greater number are so moderately remunerated as to be incapable of making provision against the emergencies of sickness and infirmitiy, otherwise than by means of some association in the nature of a friendly society. There is, consequently, a wide field for the operation of a well-devised scheme of mutual assistance such as is now projected. What nurses cannot singly accomplish they can attain by co-operation. With a well-calculated scale of contributions they may secure themselves against the effects both of sickness and of old age, though either might prove calamitous if they depended solely on their own individual resources. It is believed that when nurses have organised themselves into a self-supporting institution others will come forward with donations and subscriptions to supplement the moderate degree of provision which they are able to make. If this part of the scheme re alises what may be fairly expected of it, the self-supporting portion wil l be far from representing the whole of the benefit to which members may look forward, in respect either to the amount they receive or to the terms on which it is obtained. The managers of hospitals are likely to be among the first to recognise the claims of the institution. When persons engaged in the service of other public bodies are req uired to furnish the security of a guarantee society, it is customary for their employers to pay the premiums. The case of nurses is sim ilar, only they have to insure health instead of honesty, and, as they are constantly exposed to infection, it would be no great stretch of liberality if they were provided by the hospital funds with insu rance against the risk of sickness and infirmity. We may be confident, at any rate, that the matter will be carefully considered by hospital committees ; and, as to the assistance to be anticipated from the general public, it will be contrary to all past experience in connection with medical charities if the appeal fails to meet with a generous response.

### The Aberdeen Evening Gazette.

An excellent project.

### The Dublin Express.

The time is opportune for considering a proposal for establishing a National Pension Fund for Nurses and Hospital Officials. It may surprise our readers to learn that no such provision has been made for a class of public servants to whom humanity is so much indebted for the alleviation of suffering and the preservation of life. The world owes much to the kindness and skill of physicians and surgeons who devote the labour of their lives to the pursuit of a noble profession which seeks to mitigate the ills that flesh is heir to, but none are more ready than they are to admit that their best efforts would be fruitless if they had not the assistance of kind and competent nurses to carry out their remedial measures. As much depends upon the care and judgment of the nurses in critical cases, as upon the services of the most eminent doctors. They have special claims upon the generous consideration of the community; for the pursuit in which they are engaged is arduous and harassing, imposing a severe strain upon the physical powers, while it also brings into requisiti on the highest moral qualities. The courage and endurance which a good nurse exhibits in her daily life, exposed as she is to the peril of infection and to the trying ordeal of many hours of weary watching in the depr essing monotony of hospital work, claim the admiration and sympathy of the community. It is a strange omission, however, that the hospital nurses and the officials who discharge responsible duties, requiring intelligence and diligence in the management of our medical institutions, receive no share of the generous philanthropy of which there are so many splendid monuments on every side. The idea that there should be a National Fund has met with general approval, and already a large number have intimated their willingness to join. Such a fund would crown the work of training hospital nurses. The public have a deep interest in the subject, and we are sure would gladly co-operate in making provision for trained nurses, while raising and fixing the standard of fitness.

### The British Medical Journal.

A proposal which more directly concerns the welfare of the nurses themselves, is the creation or extension of a provident system upon a sound actuarial basis. It is one to which the attention of the consultative committee to be appointed by her Majesty might well be given ; for it is one which will need some fostering care in its early days ; in particular, there is wanted some authoritive appeal which shall be made to reach every training school and nurse. To be a success, such a provident society must be founded on a broad basis ; there is safety in numbers. If once the advantages which it might offer could become widely known, such a provident institution might quickly become, to a large extent, self-supporting, but there would always be scope for external assistance, and probably no more truly useful destination could be found for a part, at least, of the fund which may, perhaps, be known in future as Queen Victoria's Bounty.

### The Lancet.

Mr. Burdett has announced a benevolent proposal to make the prospects of nurses more comfortable in a financial sense. There can be but one opinion of the desirableness of such a provision, if it be well based. Mr. Burdett sees in the women's jubilee offering, and the Queen's views as to its disposal, the possible basis of such a fund as will guarantee to certificated nurses beyond the age of fifty a pension proportionate to the contributions which they have made. It is for nurses themselves, under the advice of actuaries, to say whether a special fund is better than insurance in some ordinary way, as the Post Office or the insurance offices, which guarantee the payment of a sum at a given age, on condition of regular payment over a series of years. There seems an extensive desire on the part of nurses to join in a pension scheme of their own. The gracious intention of her Majesty presupposes an organization that will command public confidence, and, at the same time, represent nurses widely and faithfully.

